# Potential for early warning of viral influenza activity in the community by monitoring clinical diagnoses of influenza in hospital emergency departments

**DOI:** 10.1186/1471-2458-7-250

**Published:** 2007-09-19

**Authors:** Wei Zheng, Robert Aitken, David J Muscatello, Tim Churches

**Affiliations:** 1 Centre for Epidemiology and Research, New South Wales Department of Health, Sydney, Australia

## Abstract

**Background:**

Although syndromic surveillance systems are gaining acceptance as useful tools in public health, doubts remain about whether the anticipated early warning benefits exist. Many assessments of this question do not adequately account for the confounding effects of autocorrelation and trend when comparing surveillance time series and few compare the syndromic data stream against a continuous laboratory-based standard. We used time series methods to assess whether monitoring of daily counts of Emergency Department (ED) visits assigned a clinical diagnosis of influenza could offer earlier warning of increased incidence of viral influenza in the population compared with surveillance of daily counts of positive influenza test results from laboratories.

**Methods:**

For the five-year period 2001 to 2005, time series were assembled of ED visits assigned a provisional ED diagnosis of influenza and of laboratory-confirmed influenza cases in New South Wales (NSW), Australia. Poisson regression models were fitted to both time series to minimise the confounding effects of trend and autocorrelation and to control for other calendar influences. To assess the relative timeliness of the two series, cross-correlation analysis was performed on the model residuals. Modelling and cross-correlation analysis were repeated for each individual year.

**Results:**

Using the full five-year time series, short-term changes in the ED time series were estimated to precede changes in the laboratory series by three days. For individual years, the estimate was between three and 18 days. The time advantage estimated for the individual years 2003–2005 was consistently between three and four days.

**Conclusion:**

Monitoring time series of ED visits clinically diagnosed with influenza could potentially provide three days early warning compared with surveillance of laboratory-confirmed influenza. When current laboratory processing and reporting delays are taken into account this time advantage is even greater.

## Background

Early detection is crucial for achieving effective control of outbreaks and epidemics of influenza. Monitoring of clinical and other health data streams which are available electronically in real-time or near real-time is now recognised internationally, nationally and locally as an essential complement to established mechanisms for public health surveillance [1-4]. Several studies have explored the potential utility of surveillance using various data streams for influenza monitoring [5-12]. Syndromic surveillance using emergency department (ED, or 'emergency room') data streams is becoming a popular method of monitoring disease activity [13-19].

While some studies have evaluated syndromic surveillance against continuous laboratory series [20,21], none have assessed the timeliness of ED-based syndromic surveillance against such a standard. Furthermore, few studies have analysed the more fine-grained daily temporal relationship between a syndromic and laboratory time series.

In 2001, the State of New South Wales (NSW), Australia, which has a population of approximately seven million, mandated the reporting of positive laboratory test results for influenza virus to the Department of Health [22]. In 2003, the NSW Department of Health established a near real-time ED-based syndromic surveillance system, which includes patient visits with influenza as the primary provisional diagnosis amongst the syndromes able to be monitored on a daily basis [17]. Counts of ED presentations for influenza syndrome from this system form part of an enhanced surveillance effort during the annual influenza season [23].

Amongst the many well-established analytical techniques for time series, cross-correlation analysis is frequently used to quantify the temporal relationship between two time series and to assess the statistical significance of their correlation at various time lags or offsets. However, before undertaking such analysis, care must be taken to remove both long-term trend and autocorrelation from the time series, lest they give rise to spurious evidence of a temporal relationship between data series [24,25]. Autocorrelation refers to non-independence of counts over time. The danger of ignoring these temporal confounders has long been recognised [26,27]. As Bowie and Prothero note, "cross-correlating two series both exhibiting seasonality before eliminating seasonal and trend components will inevitably produce highly significant correlation coefficients, in fact, no direct association may exist." [26]. They go on to illustrate this by demonstrating a substantial (r = 0.67) and statistically significant correlation co-efficient for time series of deaths each month due to ischaemic heart disease in the UK and the monthly tonnage of oranges imported into Britain. However, after removal of trend and seasonal components from these clearly unrelated time series, no significant cross-correlation remains.

The purpose of this paper is to investigate, with adequate control of temporal confounders, whether daily counts of ED influenza visits provided by a near real-time syndromic surveillance system offers the potential for earlier warning of changes in influenza activity in the community than traditional laboratory-based surveillance, and to estimate the magnitude of any time advantage.

## Methods

### Data sources

#### Emergency Department data

For the period 1 January 2001 to 31 December 2005, we obtained from the NSW Emergency Department Data Collection (EDDC) [28] the time series of daily counts of ED visits that were assigned a provisional diagnosis of influenza by medical staff, aggregated by date of arrival at the ED. The EDDC collects data that are routinely captured in the ED clinical information systems of 61 urban and large regional public hospitals across NSW, covering approximately three quarters of all ED visits in the state [29]. Public hospitals provide almost all ED services in NSW. Only data from the 49 EDs that contributed data continuously over the entire five-year period were included in this analysis. We have no reason to believe that this selection on the basis of time series completeness introduced any biases into the pooled count data, as the EDs that have participated continuously represent the majority of urban and larger rural EDs. The EDDC was used as the retrospective data source rather than the current near real-time ED surveillance system because of its greater temporal and geographic coverage – the near real-time ED surveillance system has been in operation only since September 2003 and currently covers only 30 EDs. However, both systems draw data from the same sources and are therefore identical with respect to the data items used in this study. International Classification of Diseases (ICD) version nine code 487 or version ten codes J10 and J11 were used to select records. The time series of this diagnosis has previously been found to have a qualitative similarity with laboratory diagnosed influenza [30].

#### Laboratory data

From 1 January 2001 in NSW, it became mandatory that all public and private laboratories notify the health department of all positive influenza test results. Public laboratories receive specimens from public hospitals whereas private laboratories receive them from general practitioners, private hospitals and other private health care facilities, such as aged care facilities. Notifications from private laboratories constitute the majority of the influenza notifications. For the same period as the ED time series, we obtained from the NSW notifiable disease surveillance system the time series of daily counts of laboratory-confirmed influenza cases, aggregated by date of disease onset. For the majority (98.5%) of influenza notifications, the date of disease onset is the collection date of the laboratory specimen. All sub-types of influenza virus were included. These results include direct detection of viral antigen from nasopharyngeal swabs or aspirates by direct immunofluorescence, detection of viral nucleic acid by polymerase chain reaction, serological tests and isolation of virus by culture. Multiple notifications for the same case of influenza were counted only once.

### Data analysis

Descriptive analyses were performed using SAS version 8.02 [31] and time series analysis was performed using the R statistical package version 2.2.0 [32].

### Poisson regression models

In order to remove long-term trend and autocorrelation from the ED and laboratory time series of daily counts, we fitted generalised linear regression models to each series for the full five-year period as well as each individual year. We assumed a "quasi-Poisson" error distribution for the model, which is appropriate for count data exhibiting over-dispersion (greater variance than would be expected in Poisson-distributed data). Because both ED visits and specimen collection are likely to be affected by holidays, weekends and other factors that vary by day of the week, we included terms in the models for weekday, public holidays, and school holidays. "Natural cubic smoothing splines" are recognised as an effective means of controlling for long-term trends in time series of counts and have been used in studies of the short-term effects of air-pollution [33-35]. These splines smooth a time series by fitting a pre-specified number of piecewise polynomials along the time series. The number of "knots", or end-points of the polynomials, determines the degree of smoothing. When the smoothed curve is subtracted from the original time series, the residual values will only include short-term variation of the observations because longer-term trend is captured in the spline curve. An *a priori *decision was made to choose the minimum degree of smoothing that would adequately remove autocorrelation and trend from the two five-year time series. To ensure comparability of results between years, we applied the same degree of smoothing when fitting models to the individual one-year time series. The final model had the form:

Expected(log(influenza counts)) = day-of-week + school holiday + public holiday + spline(day, degrees of freedom)

Fitted values obtained from the model were then subtracted from the original time series to leave two sufficiently "stationary" and "whitened" residual time series that allowed valid inferences to be drawn from cross-correlation analysis [24,25]. The Ljung-Box test, with a significance level of 0.05, was used to check autocorrelation in the residual time series prior to cross-correlation analysis [36].

### Cross-correlation analysis

Cross-correlation analysis calculates a series of correlation coefficients between two time series by overlaying and temporally shifting the two series over a range of successive time lags. This allows determination of the time lag that maximises the strength of the correlation between the two time series. R software automatically chooses between 20 and 30 positive and negative lags in its cross-correlation function, depending on the number of observations. Statistical significance was defined as a correlation coefficient greater than twice the standard error.

### Qualitative comparison of medium to long term trends

The requirement for a de-trended (stationary), non-autocorrelated time series for the cross-correlation analysis meant that we were unable to use statistical methods to compare longer-term trends between the ED and laboratory time series. However, a visual comparison of both the observed time series counts and the fitted spline component of the ED and laboratory models nevertheless allows an impression to be gained of the relative location, shape and magnitude of peak influenza activity between the two series.

### Comparison of smoothed raw time series

To assess the plausibility of the observed correlation, we plotted the 7-day moving averages of the raw ED series and the laboratory series for the most distinct influenza season in the five-year period, which was 2003.

### Estimate of laboratory processing and reporting delays

In a separate analysis, we used the NSW notifiable disease surveillance system to calculate the median lag in days between the date when the specimen was taken from the patient and the date the laboratory reported the positive test results to local health authorities.

This study used de-identified epidemiological information and therefore ethical approval was not required.

## Results

### Descriptive statistics

**Figure 1:**
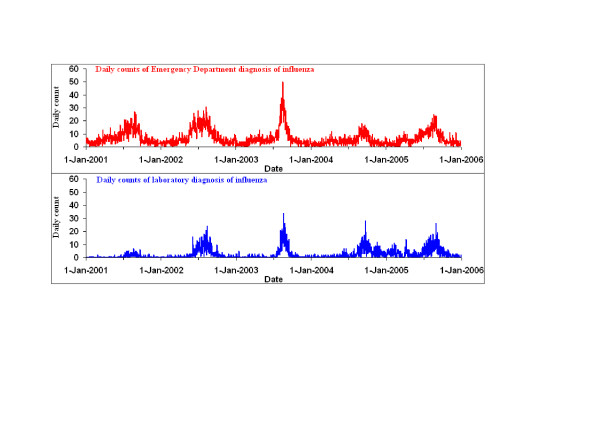
Comparison of daily count of Emergency Department diagnoses of influenza and daily counts of positive laboratory test results for influenza viruses, New South Wales, 2001 to 2005.

Visual inspection reveals broad similarity between the ED and laboratory daily series in terms of both the relative size and timing of seasonal peaks (Figure 1). The low counts early in the laboratory series probably reflect under-reporting associated with the commencement of mandatory notification of influenza by laboratories in 2001. Fourteen per cent of ED visits for influenza were by young children (ages 0–4 years) and 55 per cent were by young and middle-aged adults (ages 15–44 years). Laboratory results showed a greater predominance of young children, with 31 per cent of positive test results being for children aged under five years (Table 1). The age distribution of patients diagnosed with influenza in EDs stay fairly constant throughout the year. However, the age distribution of laboratory diagnoses of influenza differed markedly between seasons, with 40% of results for children aged 0–4 years during the annual influenza season, compared with seven per cent at other times of the year. The balance between the sexes was similar for both data sources. The severity of ED presentations with a provisional diagnosis of influenza was low, as indicated by a hospital admission rate of approximately five per cent, consistent across seasons. Almost 80% of laboratory isolates were for influenza A virus. In terms of area of residence, city and rural areas were similarly represented in the ED data and this ratio remained consistent through the course of each year. By contrast, urban residents were relatively over-represented amongst the laboratory notifications, with three-quarters living in major cities.

**Table 1:** Demographic, clinical, viral type and geographic characteristics of Emergency Department (ED) visits assigned a provisional diagnosis of influenza and laboratory-confirmed influenza notifications by season, New South Wales, 2001 to 2005

		**ED provisional diagnosis of influenza**						**Laboratory-notified influenza infection**					
		Influenza season (May-Sep)		Rest of year		**Total**		Influenza season (May-Sep)		Rest of year		**Total**	
		No.	%	No.	%	**No.**	**%**	No.	%	No.	%	**No.**	**%**
**Age group**	0–4 yrs	1108	14%	567	14%	**1675**	**14%**	1298	40%	90	7%	**1388**	**31%**
	5–9 yrs	526	7%	162	4%	**688**	**6%**	210	6%	33	3%	**243**	**5%**
	10–14 yrs	487	6%	166	4%	**653**	**5%**	162	5%	41	3%	**203**	**4%**
	15–19 yrs	825	11%	437	10%	**1262**	**11%**	118	4%	60	5%	**178**	**4%**
	20–24 yrs	930	12%	553	13%	**1483**	**12%**	102	3%	105	8%	**207**	**5%**
	25–29 yrs	710	9%	489	12%	**1199**	**10%**	108	3%	97	8%	**205**	**5%**
	30–34 yrs	705	9%	400	10%	**1105**	**9%**	128	4%	108	8%	**236**	**5%**
	35–39 yrs	558	7%	302	7%	**860**	**7%**	118	4%	93	7%	**211**	**5%**
	40–44 yrs	435	6%	281	7%	**716**	**6%**	116	4%	79	6%	**195**	**4%**
	45–49 yrs	374	5%	182	4%	**556**	**5%**	98	3%	71	6%	**169**	**4%**
	50–54 yrs	283	4%	159	4%	**442**	**4%**	94	3%	78	6%	**172**	**4%**
	55–59 yrs	215	3%	111	3%	**326**	**3%**	98	3%	79	6%	**177**	**4%**
	60–64 yrs	143	2%	95	2%	**238**	**2%**	118	4%	73	6%	**191**	**4%**
	65–69 yrs	122	2%	74	2%	**196**	**2%**	112	3%	74	6%	**186**	**4%**
	70–74 yrs	126	2%	79	2%	**205**	**2%**	101	3%	70	5%	**171**	**4%**
	75–79 yrs	93	1%	41	1%	**134**	**1%**	94	3%	57	4%	**151**	**3%**
	80–84 yrs	84	1%	34	1%	**118**	**1%**	99	3%	34	3%	**133**	**3%**
	85+ yrs	76	1%	34	1%	**110**	**1%**	101	3%	32	3%	**133**	**3%**
	Unknown	0	0%	1	0%	**1**	**0%**	0	0%	1	0%	**1**	**0%**
													
**Sex**	Male	3767	48%	2063	50%	**5830**	**49%**	1671	51%	569	45%	**2240**	**49%**
	Female	4032	52%	2104	50%	**6136**	**51%**	1601	49%	705	55%	**2306**	**51%**
													
**Admission status**	Admitted	434	6%	193	5%	**627**	**5%**	N/A	N/A	N/A	N/A	N/A	N/A
	Not Admitted	7360	94%	3966	95%	**11326**	**95%**	N/A	N/A	N/A	N/A	N/A	N/A
													
**Influenza type**	A	N/A	N/A	N/A	N/A	N/A	N/A	2635	80%	974	76%	**3609**	**79%**
	B	N/A	N/A	N/A	N/A	N/A	N/A	534	16%	235	18%	**769**	**17%**
	Both A & B	N/A	N/A	N/A	N/A	N/A	N/A	43	1%	47	4%	**90**	**2%**
	Unspecified	N/A	N/A	N/A	N/A	N/A	N/A	63	2%	19	1%	**82**	**2%**
													
**Area of residence**	Major cities	4094	52%	2209	53%	**6303**	**53%**	2495	76%	985	77%	**3480**	**76%**
	Inner regional	2452	31%	1261	30%	**3713**	**31%**	517	16%	184	14%	**701**	**15%**
	Outer regional	990	13%	546	13%	**1536**	**13%**	216	7%	83	7%	**299**	**7%**
	Remote	205	3%	81	2%	**286**	**2%**	24	1%	5	0%	**29**	**1%**
													
**Total**		**7800**	**100%**	**4167**	**100%**	**11967**	**100%**	**3275**	**100%**	**1275**	**100%**	**4550**	**100%**

Note: categories may not add to the totals due to a small proportion of unknown values.

### Poisson regression models

**Figure 2:**
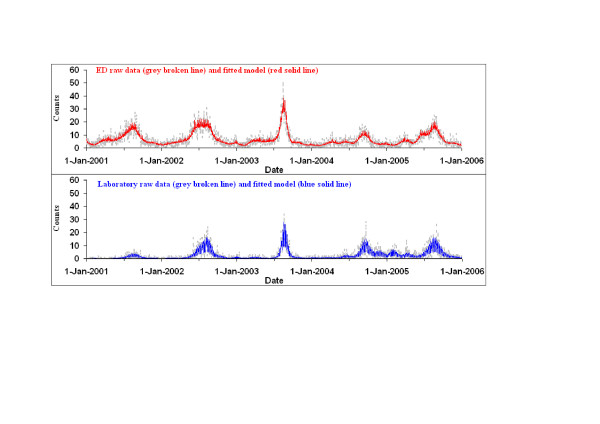
Daily counts of Emergency Department ('ED') visits with a provisional diagnosis of influenza and positive laboratory ('Lab') test results for influenza viruses – raw data and fitted models.

The specified model with eleven degrees of freedom per year (55 total degrees of freedom) was the minimum degree of smoothing that adequately limited autocorrelation within the two five-year time series. Plots of raw daily count series of both ED and laboratory data and the fitted values obtained from the Poisson regression models are shown in Figure 2. No significant autocorrelation was detected in the residual series from the models fitted to individual years, except for the ED model in 2002 and the laboratory model for 2005 (Table 2).

**Table 2:** Results of the Ljung-Box test for autocorrelation in the residual time series after removal of calendar effects and trend

		**Ljung-Box Test**	
**Year**	**Time series**	**Q-statistic**	**P-value**
2001	ED	11.72	0.07
	Laboratory model	8.29	0.22
2002	ED	14.60	0.02*
	Laboratory	5.26	0.51
2003	ED	4.36	0.63
	Laboratory	6.16	0.41
2004	ED	3.25	0.78
	Laboratory	4.25	0.64
2005	ED	3.25	0.78
	Laboratory	13.71	0.03*
2001 to 2005	ED	11.72	0.16
	Laboratory	13.58	0.09

* Indicates presence of statistically significant autocorrelation in the residual series.

### Cross-correlation analysis

**Figure 3:**
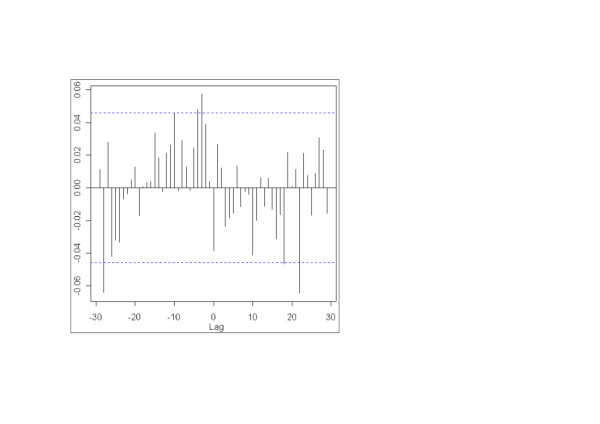
**Cross-correlation of daily counts of Emergency Department visits with a provisional diagnosis of influenza against daily counts of positive laboratory test results for influenza viruses after removal of trend and seasonality for the five-year period 2001 to 2005**. Dashed horizontal lines are 95% confidence limits.

For the full five-year period, the strongest statistically significant positive correlation (r = 0.06, p = 0.01) for the residuals of the ED model relative to the residuals of the laboratory model occurred at a lag of negative three days. This suggests that short-term changes in the ED time series preceded those in the laboratory series by three days on average (Figure 3 and Table 3). A cluster of small positive correlations (lags 2–5 days) around the largest positive correlation was evident (Figure 3). For individual years, the ED residual series was maximally significantly positively correlated with the laboratory residual series at lags ranging from -3 to -18 days (r: 0.11–0.14) (Table 3).

**Table 3:** Cross-correlations between the model residuals of the Emergency Department influenza time series and the laboratory influenza time series by year, 2001 to 2005

**Year**	**The highest and statistically significant correlation coefficient**	**P value**	**Lag of highest correlation (days)**
2001	0.12	0.02	-11
2002	0.14	0.01	-18
2003	0.12	0.03	-4
2004	0.12	0.03	-3
2005	0.11	0.02	-4
2001 to 2005	0.06	0.01	-3

### Qualitative comparison of medium to long term trends

**Figure 4:**
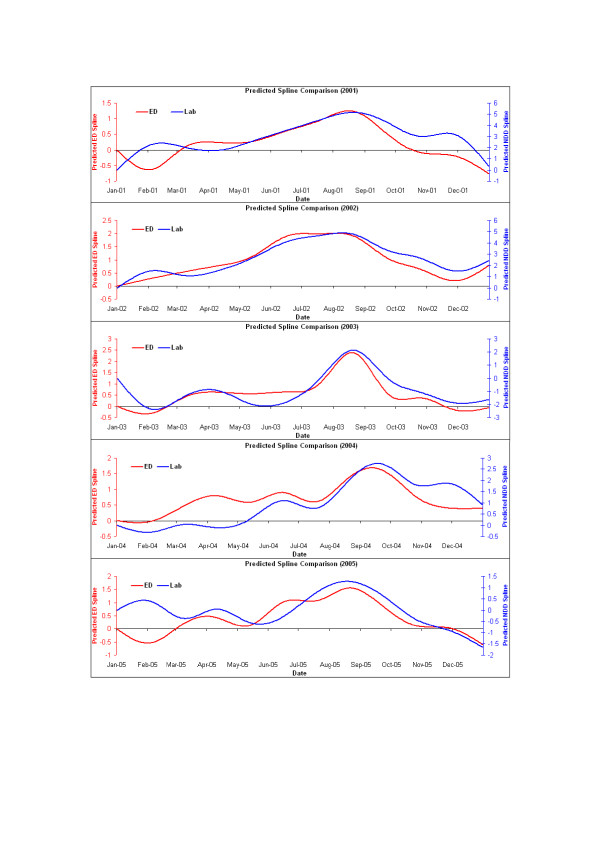
**Comparison of unlagged predicted values for the smoothing spline component of the fitted Poisson regression models, representing medium to long-term trends of the Emergency Department ('ED') and laboratory ('Lab') series by year**. Y-axis scales are chosen to give a similar range for both ED and laboratory series in each year to allow comparison of trend rather than absolute magnitude of the series over time.

Visual comparison of the logarithm of the smoothing spline component (that is, of the seasonality and longer-term trends) of the fitted models for each year showed a strong similarity in the timing and relative magnitude of influenza peaks, and a broad similarity in the overall shape of peaks between the two data sources (Figure 4). Outside of the peak influenza season some variations occurred such as in late 2001 when laboratory results tailed off more slowly than ED visits. In 2004, ED activity rose earlier in the year than laboratory results. At the end of 2004 and in early 2005 laboratory trends departed from and were higher than ED visits. It is known that during that period, the laboratory time series included some false positive results reported by a single laboratory.

### Comparison of smoothed raw time series

**Figure 5:**
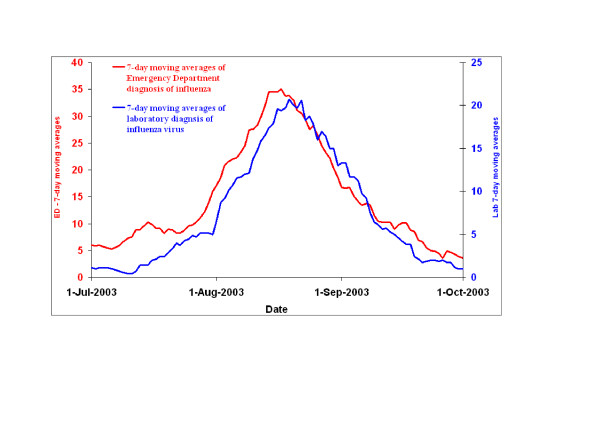
Comparison of 7-day moving averages of the Emergency Department series and 7-day moving averages of the laboratory series during the influenza season from July to September 2003.

Visual comparison of the 7-day moving averages of ED visits and laboratory results showed that during the influenza season from July to September 2003, the ED activity consistently rose, peaked and then declined earlier than the laboratory results. However, at the end of the season, the laboratory results tailed off more quickly than the ED visits (Figure 5).

### Estimate of laboratory processing and reporting delays

For the period 2001 to 2005, the median delay for the reporting of positive laboratory results for influenza to local public health authorities was four days after the date of specimen collection.

## Discussion

Through use of time series methods with adequate control of temporal confounders we found that monitoring short-term changes in the incidence of ED-diagnosed influenza could provide at least three days early warning of changing influenza activity in the population relative to monitoring short-term changes in laboratory-based surveillance data. Despite the small residual correlation coefficients, the consistency of this result across each of the most recent years and the whole five years suggests that this finding did not occur by chance. The unclear correlations in the two earlier years may have arisen from the immaturity of laboratory-based surveillance at that time. The time lag between the two data sources was clearly evident when plotting the smoothed raw incidence for the year with the most distinct seasonal influenza epidemic (2003). The close similarities between the longer-term trends within each time series provides further evidence that counts of ED visits diagnosed with influenza closely mirror the circulation of influenza virus in the population. This time advantage could be exploited through, for example, the use of statistical process control techniques, such as cumulative sum (CUSUM) or exponentially weighted moving average techniques that respond to short-term variation in the ED time series [37].

If both time series accurately represented actual incidence of influenza infection in the population then we would expect the two time series to be perfectly aligned in time. We believe, however, that the observed lag arises from the different aspects of the disease process that predominate in each data source. Respiratory specimens are more likely to be taken from patients with more advanced or more serious illness or those who are experiencing secondary complications of infection such as pneumonia. In the ED, on the other hand, otherwise healthy patients with a classic influenza-like syndrome at the early stage of illness are more likely to be assigned the provisional influenza diagnosis and are also unlikely to have a specimen taken. This would also partly explain the low correlation coefficients observed, because the exact time lag between incidence of uncomplicated and more severe illness would not be fixed but would vary according to some distribution. This is supported by the observation of a cluster of small positive correlations around the most significant lag. Other reasons for the small correlations could be the loss of information arising from the pre-whitening process, and the fact that the two data sources are drawn from quite different sources and neither can provide complete coverage of all influenza infections in the population; the ED data is from public hospital EDs, while the laboratory results could be from specimens taken in general or specialist medical practices, private or public hospitals (admitted or non-admitted patients), and nursing homes, for example. Note that nearly all EDs in our state are in public hospitals. The different age structures confirm the limited overlap between the two data sources.

If the early warning potential of ED-diagnosed influenza could be exploited using a sufficiently sensitive and specific signalling technique, the public health benefits would be manifold. It is unlikely that public health professionals would treat influenza virus as the certain cause of such a signal. However, the "situational awareness" of increased influenza-like illness activity could trigger a cascade of activities aimed at excluding more serious public health threats. Those threats could include the emergence of a mutated strain of influenza, introduction into the population of an agent, by natural or intentional means such as bioterrorism that mimics influenza in its prodromal stage, or seasonal vaccine failure. Influenza's short incubation period (around two days) and its capricious propensity to mutate underline the importance of early warning systems. The activities that might follow a signal might includes increasing the number of tests ordered for patients with influenza-like illness, applying a wider battery of tests than would otherwise be performed, and then using that information to guide subsequent public health prevention and control efforts. These activities would be particularly important outside of the influenza season when there is a low index of suspicion for influenza but when outbreaks have occurred [38]. In many countries a pandemic strain of influenza is likely to be detected independently of ED surveillance through epidemiological suspicion in travellers, but detection prior to circulation is not guaranteed. Further, pandemics often occur in waves, and should a pandemic commence, early warning of a second wave would be an advantage, particularly as laboratory testing may have declined once the pandemic is well established.

Because our analysis is effectively based on the date of specimen collection for the laboratory time series, it does not reflect the inevitable real-life delays involved in transporting specimens to laboratories, performing the tests and reporting the results to health authorities responsible for surveillance. At present in NSW, the median delay for the reporting of positive laboratory results for influenza is four days after the date of specimen collection. In the NSW ED surveillance system, where data capture is near real-time, more than three-quarters of all ED diagnoses are available for analysis within one day of the patient's arrival at the ED [17]. Thus the time advantage offered by monitoring ED visits is likely to be closer to six or seven days. There is potential, however, for laboratory delays to be reduced in future through the use point-of-care tests [39] and automated electronic reporting of results initiated as soon as test results are entered into laboratory information systems. Another option would be to monitor test orders for respiratory specimens, however, in our State year-round information on orders is not routinely reported to the Department of Health.

This study differs in several respects from previously published assessments of the timeliness of syndromic data streams. To our knowledge, no previous timeliness studies have compared daily ED visits for influenza with a continuous population-based laboratory standard. The use of daily count data in this study also allows for a more fine-grained estimation of time lags between data sources. Many syndromic surveillance systems analyse data on a daily basis, thereby giving our analysis direct relevance. Another advantage of this study is that the seasonality, longer-term trends and autocorrelation, which are inherent in population-based count data for a readily communicable disease such as influenza, were appropriately removed prior to undertaking the cross-correlation analysis. This essential step permitted valid statistical inferences to be drawn from the results, although for the ED series in 2002 and the laboratory series in 2005, autocorrelation could not be adequately controlled using our modelling strategy. We believe that autocorrelation in our data and in infectious disease time series generally derives largely from the communicability of the organism, resulting in successive incidence observations being serially correlated as the infection spreads through the population. In 2002, there was a somewhat prolonged period of seasonal influenza and this may have influenced the overall autocorrelation for the ED data for that year. This may not have been as apparent in the laboratory series because of incomplete reporting in the early years of mandatory laboratory notification. In the laboratory series for 2005, the incomplete control of autocorrelation may have arisen from a period of false positive results that are known to have been reported by one laboratory in that year.

Other analyses using daily count data have been conducted. Brownstein *et al. *reported time advantages of up to 50 days for ED-identified seasonal respiratory activity against a pneumonia and influenza mortality standard [16]. However, in that study spectral decomposition was used to filter out all but the background annual seasonal component of each time series. This technique is likely to miss the effect of influenza because the timing and severity of seasonal influenza epidemics are marked by excess pneumonia and influenza mortality over and above the seasonal background [40-42]. The seasonal background can be caused by any of the many respiratory organisms that circulate during the cooler months. Ohkusa *et al. *compared daily counts of over-the-counter sales of common cold medications to a reference standard of a national ambulatory care-based influenza database and found that the medication data provided no time advantage [10].

Other studies have used weekly counts to evaluate syndromic data streams for the surveillance of influenza. Using correct control of temporal confounders, Doroshenko *et al*. compared count data from a health advice telephone service to counts from a general practice-based sentinel surveillance system for influenza-like illness [9] and found a time advantage of between one and three weeks for the telephone service data. However, it was not clear to what extent the influenza-like illness activity data from the sentinel surveillance system reflected circulating levels of influenza virus. Other studies have compared weekly counts from a variety of syndromic data streams with, in some cases, a reference standard of laboratory-confirmed influenza, but failed to control for the confounding effects of seasonal and long-term trends or autocorrelation [5,11,12].

The data sources we used have some limitations. ED provisional diagnoses are not coded by trained clinical coders but instead are selected by ED medical and nursing staff in the course of their work. Coding practice may vary between hospitals and staff and may change over time. It is known that not all people with influenza infections are assigned the ICD codes for influenza, and there are a number of other valid but less specific codes that can be used, such as 'unspecified viral infection' or 'viraemia'. Conversely, some ED patients infected by other organisms but presenting with influenza-like symptoms could have been assigned the influenza diagnosis. Although this may have contaminated the ED series, it would be unlikely that other organisms would cause such a consistent correlation across individual years when compared with a laboratory standard. Also, Bourgeois et al. [21] demonstrated that, in children, respiratory syncytial virus (RSV), one of the most common respiratory pathogens had incidence patterns quite distinct from those of influenza in the majority of the years they studied. The similarity between the broad shapes of the two time series further supports the assertion that influenza was largely driving our ED time series. Further, clinical diagnosis of influenza in adults has been found elsewhere to be at least as accurate as either the rigorous application of a formal epidemiological case definition for influenza or many types of rapid diagnostic test kits [43].

A limitation of the laboratory diagnoses may be that they are influenced by changes in the accuracy of laboratory tests over time. Also, the propensity of clinicians to order tests may vary according to their knowledge of whether influenza is circulating or not. However, the NSW laboratory notification system is a passive surveillance system and active sampling and sentinel surveillance has declined in our state over recent years. On the other hand if clinicians know that influenza is circulating they may not choose to test because influenza has a higher probability of being the cause of illness. Also, during off-season, adults with ILI may be more likely to be tested because influenza as a cause is unexpected. Under-reporting in 2001, and, as mentioned earlier, some false positive test results from a laboratory in 2005 are known problems in the laboratory series.

One aspect of the ED surveillance data not evaluated by this study is the added surveillance dimension available from other variables captured and analysed along with ED diagnoses. For example, in NSW we include routine sub-analyses by age, sex, triage (urgency) category and discharge status variables in our daily reports. These give valuable clues to the epidemiology of the syndrome and the urgency and severity of illness in presenting patients [17]. Further evaluation is required as to whether the monitoring of the severity and other epidemiological characteristics of ED visits adds value to the assessment of the virulence of circulating influenza virus and changes in the susceptibility of the population.

## Conclusion

Our findings demonstrate that ED-based syndromic data streams can provide more timely detection of unusual influenza activity in the population. Although syndromic surveillance cannot replace laboratory surveillance, the earlier warning it provides can signal the need to increase vigilance for influenza or pathogens with an influenza-like prodrome. Even just a few days early warning may be crucial in allowing public health authorities to gain a head start in implementing interventions designed to reduce disease transmission, and delay or curtail a local outbreak or seasonal epidemic of influenza, or, potentially, the start of a pandemic caused by a novel strain of virus.

## Competing interests

The author(s) declare that they have no competing interests.

## Authors' contributions

DM conceived of the study and supervised the data analysis. WZ and RA conducted the data analysis. WZ and DM drafted the manuscript, which RA and TC edited. All of the authors read and approved the manuscript.

## Pre-publication history

The pre-publication history for this paper can be accessed here:


